# Estimation of Genetic Parameters for Reproductive Traits in Hanwoo (Korean Cattle)

**DOI:** 10.3390/ani9100715

**Published:** 2019-09-24

**Authors:** Bryan Irvine Lopez, Ju-Hwan Son, Kangseok Seo, Dajeong Lim

**Affiliations:** 1 Division of Animal Genomics and Bioinformatics, National Institute of Animal Science, Rural Development Administration, Wanju 55365, Korea; irvinelopez@korea.kr (B.I.L.); tdpro@korea.kr (J.-H.S.); lim.dj@korea.kr (D.L.); 2 Department of Animal Science and Technology, Sunchon National University, Suncheon 57922, Korea; sks@sunchon.ac.kr

**Keywords:** genetic correlation, heritability, reproductive traits, Hanwoo cattle

## Abstract

**Simple Summary:**

Due to the extensive marbling of its beef, Hanwoo (Korean native cattle) has continuously gained popularity and has become a mainstay in South Korea’s animal industry. In any beef cattle production system, reproductive performance is one of the main economic aspects taken into consideration. Therefore, genetic parameter estimates are necessary to obtain indices in order to maximize the response to selection, which in turn could lead to higher profitability. To date, knowledge on the genetic parameters for reproductive traits in Hanwoo cattle is still limited. Therefore, this study estimated the variance components, heritability, phenotypic, and genetic correlations of age at first calving (AFC), calving interval (CI), days open (DO), and gestation length (GL) of Hanwoo cattle. This was done using single-trait and multi-trait animal models. Results revealed the low heritability estimates for AFC, CI, DO, and GL in both single-trait and multi-trait models, which indicated the probable slow response of these traits due to direct selection. Moreover, phenotypic and genetic correlations varied from low to high among the reproductive traits of interest. Nevertheless, heritability estimates and genetic correlations shown in this study will prove to be vital as initial estimates are considered in the genetic improvement program of Hanwoo cattle.

**Abstract:**

Genetic parameters for the reproductive traits of Hanwoo cattle were estimated using data obtained from 15,355 cows in 92 herds across South Korea, which were inseminated from May 1997 to July 2016. An “average information” restricted maximum likelihood (REML) procedure that fit in single-trait and multi-trait animal models was used to estimate the variance components of age at first calving (AFC), calving interval (CI), days open (DO), and gestation length (GL). Results showed the low estimates of heritability for all reproductive traits from both single-trait and multi-trait models. Estimates of heritability for AFC were 0.08 and 0.10 with single-trait and multi-trait models, respectively, while the estimates of heritability using the same animal models ranged from 0.01 to 0.07, 0.01 to 0.09, and 0.10 to 0.16 for CI, DO, and GL, accordingly. While AFC showed positive genetic correlations of 0.52 and 0.46 with CI and DO, respectively, the estimates of genetic and phenotypic correlations of GL with AFC were close to zero. Moreover, phenotypic correlations of GL with CI and DO were also close to zero; however, the corresponding genetic correlations were 0.13 and –0.38 for CI and DO, respectively. These estimated variance components and genetic correlations for reproductive traits can be utilized for genetic improvement programs of Hanwoo cattle.

## 1. Introduction

Hanwoo (Korean native cattle) is a mainstay in the South Korean beef industry. Its beef is very popular for its marbled fat, tenderness, juiciness, and characteristic flavor [1]. Currently, the selection index for Hanwoo is mainly based on carcass weight, backfat thickness, eye muscle area, and marbling score [2]. To the best of our knowledge, studies related to variance components, heritability, and phenotypic and genetic correlations for reproductive traits of this South Korean native breed of cattle are still limited. 

The prevailing Hanwoo cattle breeding program mainly focuses on the genetic improvement of the carcass and meat quality traits. This is essentially due to its economic importance, in addition to its data availability and ease of analysis procedures. However, reproductive traits are also pivotal, usually with significant impacts on the economic success of production [3,4]. The low profitability in beef cattle raising is primarily attributed to fertility problems characterized by an increased number of inseminations, prolonged calving intervals, high culling rates, and the reduced productive life of cows [5]. Much work needs to be done to improve the reproductive efficiency through crossbreeding and improved management techniques. However, few efforts have been put into direct selection due to low heritability or late expression of traits in the life of the animal [6]; hence, the genetic improvement of these traits occurs at a low rate and efficiency. In view of these low heritability estimates, considerable data is needed and the best model has to be defined in order to obtain accurate estimates [7], which establishes efficient selection programs that may eventually lead to greater profitability. 

As genetic parameter estimates are essential in obtaining indices for an optimized response to genetic selection, the present study aimed to estimate the variance components, heritability, and phenotypic and genetic correlations of age at first calving (AFC), calving interval (CI), days open (DO), and gestation length (GL) of Hanwoo cattle utilizing both single-trait and multi-trait animal models.

## 2. Materials and Methods

### 2.1. Data

Data were obtained from reproductive traits of 15,355 Hanwoo cows, calved from May 1997 to July 2016, and obtained from 92 herds in nine South Korean provinces. The approval of the Animal Care and Use Committee was not necessary in this study since all data were acquired from an existing database. Four reproductive traits were analyzed including age at first calving (AFC), calving interval (CI), days open (DO), and gestation length (GL). Cows with an AFC between 540 and 1000 days, a CI between 300 and 600 days, a DO between 25 and 200 days, and a GL between 260 and 310 days were retained in the final analysis. The accompanying pedigree consisted of 74,445 individuals and was 10 generations deep. The means, standard deviations, and minimum and maximum values for AFC, CI, DO, and GL are shown in Table 1. The averages for AFC, CI, DO, and GL with their corresponding standard errors were 752.42 ± 0.52, 363.11 ± 0.15, 76.40 ± 0.15 and 286.39 ± 0.02 days, respectively. 

### 2.2. Statistical Analysis

The data were first analyzed via the ordinary least squares using the MIXED procedure from the statistical software SAS (SAS Institute, Inc., Cary, NC, USA, 2012) in order to determine the significance of fixed effects and their interactions. These fixed effects and covariates tested for significance in each trait where the herd in which the animal was raised, the parity of the cow, the year-month and year-season of birth and calving, the sex of the calf, and the age of the cow (months) were covariates. Based on preliminary analyses, herd, parity, and year-month were included as contemporary groups in the models. 

Variance components were estimated through an “average information” restricted maximum likelihood procedure, both in single- and multi-trait animal models, using the WOMBAT software [8]. To show the variance components of the permanent environmental effects of the traits and to compare the estimates at different parities, two sets of single-trait analyses were performed: (1) single-trait analysis by parity (1, 2, and 3) and (2) single-trait analysis by trait (repeatability model). The matrix representation of the general linear model is:

(1) $$y\;=Xb+Z_{1}a+Z_{2}c+e$$

where y is the vector of observations; b is the vector of fixed effects; a is the vector of direct additive genetic effects of dams; c is the vector of permanent environmental effects, included as random; and e is the vector of random errors associated with the observations. X, Z_1_, and Z_2_ are incidence matrices related to fixed, additive genetic, and permanent environmental effects, respectively. Vector a was assumed to follow a normal distribution, with N (0, A$\sigma_{a}^{2}$) where A was the relationship matrix; c was assumed to be N (0, I$\sigma_{pe}^{2}$) and uncorrelated with other random effects; and e was assumed to be N (0, I$\sigma_{e}^{2})$, where I denotes the identity matrix. Birth year-month of the dam and herd were used as fixed effects for AFC. For CI, DO, and GL, the fixed effects of calving year-month, herd, and parity were employed. The permanent environmental effects were not included in the single-trait analysis of AFC or in the analysis of CI, DO, and GL by parity. 

The convergence criterion for all runs was set to 10^−8^. The output generated from the program included (co)variance components and their corresponding standard errors. Heritability (*h*^2^) was defined as the ratio of genetic variation that is due to additive genetic variance to total phenotypic variance ($h^{2}=\sigma_{a}^{2}/\sigma_{p}^{2}$).

## 3. Results

### 3.1. Genetic Parameters

Here, we compared the heritability and variance components obtained using the single-trait model versus those of the multi-trait model. Genetic and phenotypic correlations were also estimated among the reproductive traits. Heritability and variance component estimates for AFC, CI, DO, and GL using the single-trait and multi-trait models are shown in Table 2 and Table 3, respectively. Estimates of heritability were low for all reproductive traits considered in this study for both single-trait and multi-trait analyses. 

#### 3.1.1. Single-Trait Model

Estimates of heritability for AFC was low (0.08). Differences in the estimated variances of additive genetic and residual effects for CI, DO, and GL were observed among parities. Although there were no large differences in the estimate of heritabilities for CI, DO, and GL among the note parities, the additive genetic and residual variances of CI and DO from those of parities 2 and 3 were higher than those of parity 1. Heritability estimates using the single-trait model ranged from 0.01 to 0.07, 0.01 to 0.09, and 0.10 to 0.16 for CI, DO, and GL, respectively. Permanent environmental effects only accounted for a small portion of the total variability in this analysis.

#### 3.1.2. Multi-Trait Model

Results of the heritability estimates with the multi-trait model for all reproductive traits were not significantly different from those found with the single-trait model, as shown in Table 3. Heritability estimates using the multi-trait model were 0.10, 0.03, 0.03, and 0.13 for AFC, CI, DO, and GL, respectively. Permanent environmental effects explained a higher portion of total variability in this analysis compared to the single-trait analysis. These percentages of variance from the said effects were 1%, 0.4%, and 6% for CI, DO, and GL, respectively.

### 3.2. Genetic and Phenotypic Correlations

Table 4 shows the genetic and phenotypic correlations between the reproductive traits of Hanwoo cattle. While the age at first calving showed positive genetic correlations of 0.52 and 0.46 for CI and DO, respectively, the corresponding phenotypic correlations were low, with values of 0.03 for both CI and DO. Moreover, the genetic and phenotypic correlation estimates of AFC with GL did not differ from zero. The CI and DO were highly positively correlated both phenotypically and genetically, as expected. Phenotypic correlations of GL with CI and DO were low, with values of −0.02 and −0.04; however, the corresponding genetic correlations were 0.13 and −0.38 for CI and DO, respectively.

## 4. Discussion

In succeeding sections, only the results from the multi-trait animal model were compared to the estimates from the related literature, owing to the fact that: (1) the present study did not involve the differentiation of the genetic parameter estimates from different models and (2) the estimated heritability for all the reproductive traits were similar in single-trait and multi-trait models.

### 4.1. Heritability Estimates

The heritability estimates for reproductive traits attained in this study were low because of a large, unexplainable portion of residual variance. Even though studies related to the estimates of variance components and heritability for reproductive traits of Hanwoo cattle are scarce, the observed low heritability estimates were comparable with previously reported estimates in other beef cattle breeds. Cavani et al. [3] and Buzanskas [9] reported low heritability estimates for AFC of 0.10 in Brahman cattle and 0.04 in Canchim beef cattle. In Nellore cows, heritability estimates of 0.11 [10], 0.17 [11], 0.25 [12], and 0.36 [4] for AFC were described in various previous works. Van der Westhuizen et al. [13] reported a moderate heritability estimate of 0.40 for AFC in multi-breed beef cattle herd. 

A number of associated studies have shown heritabilities of a similar magnitude in beef breeds for CI, DO, and GL. Using the Bayesian procedure, Ulhôa et al. [4] reported an estimate of heritability averaging 0.05, 0.04, and 0.10 for CI, DO, and GL, respectively, in Nellore cows. Moreover, Wasike et al. [14] estimated heritability by parity in Boran beef cattle, with values of 0.00, 0.15, and 0.00 for CI in the first, second, and third parities, accordingly. Oyama et al. [15] reported similar estimates of heritability for CI and DO and a higher estimate of 0.40 for GL in Japanese Black (Wagyu) cattle. Differences in estimates of heritability for GL may have occurred due to differences in the structure of the populations used, as well as non-identified environmental factors.

Therefore, the results of this present work indicated that these traits of interest would be expected to respond gradually to direct selection. This was in concordance with Aby et al. [16], who emphasized that the genetic potentials of herds for reproductive traits could be slowly enhanced through selection over the years. The availability of genomic information in the genetic evaluation of animals has provided an opportunity to enhance the efficiency of selection, especially for lowly heritable traits.

### 4.2. Genetic and Phenotypic Correlations

The genetic and phenotypic correlations between DO and CI were unsurprisingly high and positive. Hereafter, all correlations involving DO were similar to those involving CI. The moderate and positive genetic correlation of AFC with CI found in this study implies that animals that deliver their first calf earlier tend to have a reduced calving interval. However, these genetic correlations between AFC and CI presented high standard errors, which means that no reliable conclusion can be drawn from the relationships of these traits. Several reports described variations in the genetic correlations between these traits. Both Oyama et al. [15] and Berry and Evans [17] revealed the positive genetic correlations of 0.25 and 0.22 between AFC and CI for Japanese Black (Wagyu) cattle and *Bos Taurus* cattle, respectively. In contrast, Cavani et al. [3] and Mercadante et al. [18] reported low negative genetic correlations of −0.13 for Brahman cattle and −0.06 for Nellore cattle, respectively. Given the abovementioned discrepancies, further research into the genetic association between these traits is needed. 

The genetic correlation of 0.04 between GL and AFC was in agreement with other preceding studies [4,15]. Gestation length was estimated to have moderate negative genetic correlations with DO and low positive correlations with CI. These results were akin to those reported by Ulhôa et al. [4] in Nellore cows. Moreover, Oyama et al. [15] described genetic correlations of 0.16 and –0.11 for GL with CI and DO, respectively. In Fogera cattle, Bekele et al. [19] obtained a high positive genetic correlation estimate of 0.72 between GL and CI, and high negative genetic correlation of −0.94 between GL and DO. Despite some favorable genetic correlations between traits, selection may not yield substantial gains for these traits due to low heritabilities.

## 5. Conclusions

The selection of Hanwoo cattle for reproductive traits such as AFC, CI, GL, and DO may render low magnitudes and long-term responses. Nevertheless, the economic importance of these traits should not be overlooked. Therefore, these reproductive qualities should be further developed and improved so that they can reach their optimal levels through enhanced management techniques. Further, data that records direct measures of these traits for implementation of genetic prediction should be improved. The variance component estimates and genetic correlations for reproductive traits obtained in this study can be utilized for future genetic improvement programs concerning of Hanwoo cattle.

## Figures and Tables

**Table 1:** Descriptive statistics of the reproductive traits of Hanwoo cows used in this study.

Trait	N	Mean	SD	Min	Max
AFC	15,355	752.42	68.34	555	961
CI	32,599	363.11	28.74	311	450
DO	32,465	76.40	28.22	25	163
GL	49,748	286.39	5.56	270	303

AFC: age at first calving; CI: calving interval; DO: days open; GL: gestation length.

**Table 2:** Variance components and heritability estimates for reproductive traits of Hanwoo cattle at different parities using the single-trait model.

Parity	Trait	N	$\sigma_{a}^{2}$	$\sigma_{pe}^{2}$	$\sigma_{e}^{2}$	$\sigma_{P}^{2}$	h^2^	pe^2^
1	AFC	15,355	345.11	-	3861.27	4206.38	0.08 ± 0.01	-
	CI	1936	1.01	-	153.28	154.29	0.01 ± 0.05	-
	DO	1726	2.70	-	145.05	147.75	0.02 ± 0.05	-
	GL	16,039	3.82	-	24.32	28.14	0.14 ± 0.02	-
2	CI	11,144	28.59	-	708.72	737.31	0.04 ± 0.02	-
	DO	7308	53.07	-	560.74	613.81	0.09 ± 0.02	-
	GL	12,461	3.13	-	24.04	27.18	0.12 ± 0.02	-
3	CI	8201	48.01	-	635.63	683.63	0.07 ± 0.03	-
	DO	5888	50.74	-	624.66	675.40	0.08 ± 0.03	-
	GL	8953	4.20	-	21.90	26.10	0.16 ± 0.03	-
1 to ≥3	CI	32,599	14.09	0.00	680.21	694.29	0.02 ± 0.01	0.00 ± 0.01
	DO	32,465	16.31	0.00	677.42	693.73	0.02 ± 0.01	0.00 ± 0.01
	GL	49,748	3.98	0.14	27.51	23.38	0.14 ± 0.01	0.01 ± 0.01

$\sigma_{a}^{2}$: additive genetic variance; $\sigma_{pe}^{2}$: variance due to permanent environmental effects; $\sigma_{e}^{2}$: residual variance; $\sigma_{p}^{2}$: total variance; h^2^: heritability; pe^2^: fraction of variance due to permanent environmental effect.

**Table 3:** Variance components and heritability estimates for reproductive traits of Hanwoo cattle using multi-trait model.

Trait	$\sigma_{a}^{2}$	$\sigma_{pe}^{2}$	$\sigma_{e}^{2}$	$\sigma_{P}^{2}$	h^2^	pe^2^
AFC	427.78	-	3700.32	4128.10	0.10 ± 0.01	-
CI	19.80	6.73	736.41	762.94	0.03 ± 0.01	0.01 ± 0.01
DO	23.34	3.31	730.36	757.01	0.03 ± 0.01	0.004 ± 0.01
GL	3.59	1.57	22.47	27.63	0.13 ± 0.02	0.06 ± 0.01

**Table 4:** Genetic (above diagonal) and phenotypic (below diagonal) correlations among reproductive traits of Hanwoo cattle.

Trait	AFC	CI	DO	GL
**AFC**		0.52 ± 0.13	0.46 ± 0.11	0.04 ± 0.05
**CI**	0.03 ± 0.01		0.87 ± 0.04	0.13 ± 0.11
**DO**	0.03 ± 0.01	0.93 ± 0.001		−0.38 ± 0.10
**GL**	0.09 ± 0.01	−0.02 ± 0.01	−0.04 ± 0.01	

## References

[1] Jo C., Cho S., Chang J., Nam K. (2012). Keys to production and processing of Hanwoo beef: a perspective of tradition and science. Anim. Front..

[2] Kim S., Alam M., Park M.N. (2017). Breeding initiatives for Hanwoo cattle to thrive as a beef industry – A review study. J. Anim. Breed. Genom..

[3] Cavani L., Garcia D.A., Carreño L.O.D., Ono R.K., Pires M.P., Farah M.M., Ventura H.T., Millen D.D., Fonseca R. (2015). Estimates of genetic parameters for reproductive traits in Brahman cattle breed1. J. Anim. Sci..

[4] Ulhôa Magnabosco C., Brito Lopes F., de Magalhaes Rosa G.J., Sainz R.D. (2016). Bayesian estimates of genetic parameters for reproductive traits in Nellore cows raised on pasture in tropical regions. Rev. Colom. Cienc. Pecuari..

[5] Espinoza J.L., González Peña D., Palacios Espinoza A., Ortega R., Guillén A. (2016). Genetic parameters of days open in Charolais cattle of Cuba. Rev. Colom. Cienc. Pecuari..

[6] Cammack K., Thomas M., Enns R. (2009). Reproductive traits and their heritabilities in beef cattle. Prof. Anim. Sci..

[7] Lopez B.I., Kim T.H., Makumbe M.T., Song C.W., Seo K.S. (2017). Variance components estimation for farrowing traits of three purebred pigs in Korea. Asian-Australas. J. Anim. Sci..

[8] Meyer K. (2007). WOMBAT—A tool for mixed model analyses in quantitative genetics by restricted maximum likelihood (REML). J. Zhejiang Univ. Sci. B.

[9] Buzanskas M.E., Grossi D.A., Baldi F., Barrozo D., Silva L.O.C., Torres Júnior R.A.A., Munari D.P., Alencar M.M. (2010). Genetic associations between stayability and reproductive and growth traits in Canchim beef cattle. Livest. Sci..

[10] Kluska S., Olivieri B.F., Bonamy M., Chiaia H.L.J., Feitosa F.L.B., Berton M.P., Peripolli E., Lemos M.V.A., Tonussi R.L., Lôbo R.B. (2018). Estimates of genetic parameters for growth, reproductive, and carcass traits in Nelore cattle using the single step genomic BLUP procedure. Livest. Sci..

[11] Caetano S.L., Savegnago R.P., Boligon A.A., Ramos S.B., Chud T.C.S., Lôbo R.B., Munari D.P. (2013). Estimates of genetic parameters for carcass, growth and reproductive traits in Nellore cattle. Livest. Sci..

[12] Buzanskas M.E., Pires P.S., Chud T.C.S., Bernardes P.A., Rola L.D., Savegnago R.P., Lôbo R.B., Munari D.P. (2017). Parameter estimates for reproductive and carcass traits in Nelore beef cattle. Theriogenology.

[13] Van der Westhuizen R., Schoeman S., Jordaan G., Van Wyk J. (2001). Genetic parameters for reproductive traits in a beef cattle herd estimated using multitrait analysis. S. Afr. J. Anim. Sci..

[14] Wasike C.B., Indetie D., Ojango J.M.K., Kahi A.K. (2009). Direct and maternal (co)variance components and genetic parameters for growth and reproductive traits in the Boran cattle in Kenya. Trop. Anim. Health Pro..

[15] Oyama K., Katsuta T., Anada K., Mukai F. (2004). Genetic parameters for reproductive performance of breeding cows and carcass traits of fattening animals in Japanese Black (Wagyu) cattle. Anim. Sci..

[16] Åby B., Aass L., Sehested E., Vangen O. (2012). Effects of changes in external production conditions on economic values of traits in Continental and British beef cattle breeds. Livest. Sci..

[17] Berry D.P., Evans R. (2014). Genetics of reproductive performance in seasonal calving beef cows and its association with performance traits. J. Anim. Sci..

[18] Mercadante M.E.Z., Lôbo R.B., Oliveira H.N.D. (2000). Estimativas de (co) variâncias entre características de reprodução e de crescimento em fêmeas de um rebanho Nelore. Rev. Bras. Zootec..

[19] Bekele A., Wuletaw Z., Haile A., Gizaw S., Mekuriaw G. (2017). Genetic parameters for reproduction traits and correlation with pre weaning growth traits of Fogera cattle at Metekel ranch, north west Ethiopia. Livest. Res. Rural. Dev..

